# Maternal supplementation with a casein hydrolysate and yeast beta-glucan from late gestation through lactation improves gastrointestinal health of piglets at weaning

**DOI:** 10.1038/s41598-022-20723-5

**Published:** 2022-10-18

**Authors:** Alison Dowley, John V. O’Doherty, Anindya Mukhopadhya, Eadaoin Conway, Stafford Vigors, Shane Maher, Marion T. Ryan, Torres Sweeney

**Affiliations:** 1 School of Agriculture and Food Science, University College Dublin, Belfield, Dublin 4, Ireland; 2 School of Veterinary Medicine, University College Dublin, Belfield, Dublin 4, Ireland; 3 Food for Health Ireland Technology Centre, University College Dublin, Belfield, Dublin 4, Ireland

**Keywords:** Immunology, Microbiology, Molecular biology

## Abstract

Improving maternal nutrition during pregnancy/lactation is a promising strategy to maximise the intestinal health of piglets undergoing abrupt weaning under commercial production conditions. This experiment investigated the effects of maternal supplementation of a casein hydrolysate and yeast β-glucan (CH-YBG) from day 83 of gestation until weaning (day 28) on sow faecal microbial populations and measures of piglet gastrointestinal health parameters at weaning. Sows (n = 10 sows/group) were assigned to: (1) control diet, and (2) control diet + CH-YBG. Maternal supplementation increased the abundance of the phylum Firmicutes, including members *Lactobacillus* in the sows faeces, with a concomitant increase in the caecal abundance of *Lactobacillus* in the weaned piglets compared to the controls*.* Piglets weaned from the supplemented sows had increased villus height in the duodenum (P < 0.05) and increased villus height to crypt depth ratio in the jejunum, as well as a decreased expression of the proinflammatory cytokine genes (*IL6*/*TNF*/*TGFB)*, the tight junction gene *CLDN3* and the mucin gene *MUC2* in the duodenum/jejunum compared to the controls (P < 0.05). In conclusion, maternal CH-YBG supplementation during pregnancy/lactation improved microbial, structural, and inflammatory measures of gastrointestinal health of piglets at weaning. This is a promising strategy to alleviate the challenges that occur with early abrupt weaning in commercial pig production.

## Introduction

Commercial pigs are subjected to significant levels of stress at weaning. This causes an initial reduction in feed intake, contributing to adverse gut morphological and functional changes such as villous atrophy and crypt hyperplasia. This in turn, increases epithelial permeability and inflammation^1,2^. Alongside an immature immune system, these factors favour the proliferation of pathogenic bacteria, including, enterotoxigenic *Escherichia coli,* thus increasing overall susceptibility to infection and post-weaning diarrhoea^3^. Supplementation of dietary zinc oxide (ZnO) at pharmacological doses improves growth and controls the proliferation of pathogenic bacteria post-weaning. However, the EU have begun the phasing out of pharmacological doses of ZnO (Commission Implementing Decision of 26.6.2017, C (2017) 4, 529 Final) alongside further restrictions on the use of infeed medication (Regulation (EU) 2019/6) in weaner pig diets by 2022 due to their association with antimicrobial resistance.

Recent research regarding the enhancement of performance and gut health, in pigs, has focussed on post-weaning dietary manipulation. However, a more effective alternative may be supplementing the diet of the gestating and/or lactating sow to enhance the pre-weaning health status of the piglet as this may have a positive influence on post-weaning performance^4,5^. The gastrointestinal tract (GIT) of the piglet is colonized immediately after birth by bacteria originating from both the sow and the environment, and these microbes play a fundamental role in the development of a healthy GIT and immune system^6^.

The digestion or enzymatic hydrolysis of milk proteins has been shown to yield physiologically important bioactive peptides that have a wide range of biological activities^7,8^. Casein hydrolysates reduced inflammation in an ex-vivo model^9^, however in-vivo they are susceptible to breakdown in the stomach. To prevent this and preserve their bioactivity, hydrolysates can be naturally encapsulated with a yeast β-glucan. The combination of 5 kDa retentate (5kDaR), derived from moderate degree of hydrolysis (11–16%) of bovine milk casein with a yeast β-glucan in the weaning pig diet was associated with an improvement in overall performance and gut health parameters^10^. As well as acting as a microencapsulating agent, β-glucans have been extensively studied for their antioxidant, immunological, and anti-inflammatory effects^11–13^. Thus, in the present study, it was hypothesised that maternal supplementation with a casein hydrolysate + yeast β-glucan (CH-YBG) from day 83 of gestation would alter the GIT microbiota of the sow and modulate microbial populations, the inflammatory response and aspects of intestinal health of her progeny at weaning. Thus, the objective of this experiment is to investigate the effects of maternal dietary supplementation of a CH-YBG from day 83 of gestation until weaning (day 28) on sow faecal microbial populations, and measures of piglet gastrointestinal health parameters at weaning.

## Materials and methods

### Experimental design and diets

A total of 20 crossbred pregnant gilts (Large White X Landrace genetic lines) were randomly assigned to one of the two dietary groups: (T1) basal gestation and lactation diet (control) and (T2) basal gestation and lactation diet supplemented with a combination of a CH-YBG from day 83 of gestation until weaning (day 28). The gestation and lactation diet were top-dressed each morning (09.00 h) with the experimental supplement (2 g/day; 1 g casein hydrolysate + 1 g yeast β-glucan) to ensure consumption. The casein hydrolysate (CH) used in the current study was produced from the hydrolysis of sodium caseinate (NaCas, ≈ 90% w/w protein, Kerry Food Ingredients, Listowel, Ireland) derived from bovine and has been reported previously by Mukhopadhya et al.^9^. The yeast β-glucan was derived from *Saccharomyces cerevisiae* (Biothera Pharmaceuticals, Inc., Eagan, MI, USA) and the optimum concentrations of a casein hydrolysate and a yeast β-glucan (CH-YBG) were established from previous studies^14,15^. The gestation diet contained 140 g/kg of crude protein (CP), 13.5 MJ/kg of digestible energy (DE) and 4.4 g/kg of standardised ileal digestible (SID) lysine. The lactation diet contained 190 g/kg of CP, 14.5 MJ/kg of DE and 8.5 g/kg of SID lysine. All amino acid requirements were met relative to lysine^16^. The ingredient composition of the diets is presented in Table 1.

**Table 1:** Ingredient composition of the experimental diets.

	Gestation diet^a^	Lactation diet^a^
**Ingredients (g/kg unless otherwise stated)**		
Wheat	303.8	352.5
Barley	300.0	300.0
Soyabean meal	67.0	182.0
Dried maize distillers grains	60.0	100.0
Soya hulls	70.0	
Beet pulp	100.0	
Soya oil	70.0	30.0
Vitamins and minerals^b^	3.0	2.5
Salt	3.0	5.0
Dicalcium phosphate	11.2	12.0
Limestone	12.0	12.0
l-Lysine HCl		2.0
dl-Methionine		1.0
l-Threonine		1.0
**Analysis of chemical composition**		
Dry matter	870.9	873.2
Crude protein (N × 6.25)	140.0	190.0
Gross energy (MJ/kg)	16.9	17.1
Ash	55.2	57.1
Digestible energy (MJ/kg)^c^	13.5	14.5
Lysine^c^	5.5	10.0
Methionine and cysteine^c^	3.3	6.0
Threonine^c^	3.85	7.0
Tryptophan^c^	1.0	1.8
Calcium^c^	8.7	9.3
Phosphorus^c^	5.0	5.2

^a^Treatments: (1) basal diet (2) basal diet supplemented with 2.0 g casein hydrolysate + yeast β-glucan/day.

^b^Sow diet provided (per kg diet): 250 mg choline chloride; 140 mg Fe; 120 mg Zn as ZnO; 67 mg α-tocopherol; 47 mg Mn as MnO; 25 mg Cu as CuSO4; 12 mg nicotinic, acid; 10 mg pantothenic, acid; 4 mg phytylmenaquinone; 2 mg riboflavin; 2 mg thiamin; 1.8 mg retinol; 0.6 mg iodine as calcium iodate on a calcium sulphate/calcium carbonate carrier; 0.3 mg Se as sodium selenite; 0.025 mg cholecalciferol; 0.015 mg pyridoxine; 0.01 mg cyanocobalamin.

^c^Calculated for the tabulated nutritional composition^17^.

From day 83 of gestation until day 110, the gilts were housed in groups of ten and the experimental supplement (CH-YBG) was top-dressed on the gestation diet prior to feeding each morning (09.00 h) to ensure consumption. The gilts were housed in individual stalls at feeding. The gilts received 2.5 kg/day of gestation diet from day 83 until day 110 of gestation. From day 110, gilts received specific amounts of feed in the following quantities: 2 kg/day of diet from day 110 of gestation until the day of farrowing (day 0) and then the feed supply was increased by 1 kg/day until day 3 post-farrowing and by 0.5 kg/day until day 6 post-farrowing. Afterwards, the sows were allowed semi-ad libitum consumption of the standard lactation diet, which was adjusted for each sow depending on daily intake until weaning at day 28. The sows were fed in 2 equal meals provided at 09.00 and 15.00 h.

The sows and piglets were individually housed in farrowing pens (2.2 × 2.4 m) with crates, slatted floors, and heat pads for piglets. The farrowing room temperature was maintained at 20 °C. The sows were individually fed and had ad libitum access to drinking water throughout the experimental period.

On the expected farrowing date, fresh sow faecal samples (approximately 10 g) were collected from the gilts into sterile containers (Sarstedt, Wexford, Ireland) and immediately frozen (  20 °C) for subsequent 16 s rRNA sequencing.

### Management of piglets

All farrowings were supervised. Between 6 and 12 h after the birth of the last piglet, litter size was adjusted by cross-fostering piglets within sow diets to ensure that sows nursed a similar number of piglets (n = 12), and this was maintained throughout the suckling period until weaning. The piglets body weight (BW) was recorded at birth and weaning, and the average daily gain (ADG) calculated. The piglets received an intramuscular injection of Fe-dextran (Ferdex 100; Medion Farma Jaya) on day 7 after birth. No creep feed was offered to the piglets throughout the lactation period, and piglets did not have access to the sows’ feed.

### Collection of piglet tissue and digesta samples at weaning

On the day of weaning (day 28), one male piglet per sow of average birth weight (which was identified at parturition) was euthanized after a lethal injection with pentobarbitone sodium, Euthatal solution, 200 mg/ml, (Merial Animal Health, Essex, UK) at a rate of 0.71 ml/kg BW to the cranial vena cava to humanely euthanize the animal. Euthanasia was performed by a competent person in a separate room away from sight and sound of the other pigs. The entire intestinal tract was immediately removed. Sections from the duodenum (located 10 cm distal from the stomach), the jejunum (60 cm from the stomach) and the ileum (15 cm from the caecum) were excised and fixed in 10% neutral-buffered formalin. Digesta from the caecum and colon was collected and stored in sterile containers (Sarstedt, Wexford, Ireland) and immediately frozen at − 20 °C for subsequent 16 s rRNA sequencing. In addition, tissue samples were taken from the duodenum, jejunum and ileum for the purpose of measuring the expression of cytokines, digestive enzymes, nutrient transporters, mucins and tight junctions using QPCR. Tissue sections (1 cm) from the duodenum, jejunum, and ileum were cut out, dissected along the mesentery, emptied, and rinsed using sterile phosphate buffered saline (Oxoid, Hampshire, UK). The tissue sections were stripped of the overlying smooth muscle before storage in RNAlater^®^ (5 ml) solution (Applied Biosystems, Foster City, CA, USA) overnight at 4 °C. The RNAlater^®^ was removed before storing the samples at − 80 °C.

### Gut morphological analysis

Preserved duodenal, jejunal and ileal tissue samples were prepared using standard paraffin embedding techniques as previously described^18^. All samples were sectioned at a thickness of 5 μm and stained with haematoxylin and eosin. The villus height (VH) and crypt depth (CD) were measured in the stained sections (4× objective) using a light microscope fitted with an image analyser (Image-Pro Plus; Media Cybernetics, Oxon, UK). Measurements of fifteen correctly orientated and intact villi and crypts were taken for each segment. The VH was measured from the crypt-villus junction to the tip of the villus, and CD was measured from the crypt-villus junction to the base.

### Gene expression in the small intestine

#### RNA extraction and cDNA synthesis

The total RNA was extracted from the duodenal, jejunal and ileal tissue using the TRI Reagent (Sigma-Aldrich, St. Louis, MO, USA) according to the manufacturer’s instructions. This crude RNA extract was further purified using the GenElute™ Mammalian Total RNA Miniprep kit (Sigma-Aldrich) which incorporated a DNase step using an on-column DNase 1 Digestion set (Sigma-Aldrich). The total RNA was quantified using a Nanodrop-ND1000 spectrophotometer (Thermo Scientific) and the purity was assessed from the ratio of the absorbance at 260 nm and 280 nm. The RNA integrity was assessed using an Agilent 2100 Bioanalyzer using an RNA 6000 Nano LabChip kit (Agilent Technologies, Santa Clara, CA, USA). All samples had a 260:280 ratio > 2.0 and an RNA integrity number (RIN) > 8.0. The total RNA (2 μg) was reverse transcribed using a High-Capacity cDNA Reverse Transcription Kit (Applied Biosystems) and oligo (dT) primers in a final reaction volume of 40 μl, according to the manufacturer’s instructions. The cDNA was then adjusted to a volume of 360 μl with nuclease-free water.

#### Quantitative real-time Polymerase Chain Reaction (QPCR)

The quantitative PCR (QPCR) reaction mix (20 μl) contained GoTaq qPCR Master Mix (10 μl) (Promega, Madison, WI), forward and reverse primers (1.2 μl) (5 μM), nuclease-free water (3.8 μl) and cDNA (5 μl). All QPCR reactions were performed in duplicate on the 7500 ABI Prism Sequence detection System (Applied Biosystems, Foster City, CA). The cycling conditions included a denaturation step of 95 °C for 10 min followed by 40 cycles of 95 °C for 15 s and 60 °C for 1 min. All primers were designed using the Primer Express Software (Applied Biosystems, Foster City, CA) and synthesised by MWG Biotech UK Ltd (Milton Keynes, UK) and are presented in Table 2. Dissociation curves were generated to confirm the specificity of the resulting PCR products. The QPCR assay efficiencies were established by plotting the cycling threshold (CT) values derived from fourfold serial dilutions of cDNA against their arbitrary quantities and only assays exhibiting 90–110% efficiency and single products were used in this study. Normalised relative quantities were obtained using the qbase PLUS software (Biogazelle, Ghent, Belgium) from stable reference genes; *B2M*, *ACTB* and *PPIA*. These genes were selected as reference genes based on their M value (< 1.5) generated by the GeNorm algorithm within GeNorm. The genes analysed in the current study are as follows: *SLC15A1* (previously known as *PEPT1*); *FABP2*; *SLC2A1* (previously known as *GLUT1*); *SLC5A1* (previously known as *SGLT1*); *SLC2A2* (previously known as *GLUT2*); *SLC2A5* (previously known as *GLUT5*); SLC16A; SLC1A4; SLC5A8; *CCK*; *TNF*; *CXCL8* (previously known as *IL8*); *IL6*; *IL10*; *IFNG*; *MUC2*; *TGFB1*; *IL17; CLDN3*; *MUC1*; *B2M*; *ACTB*; *PPIA*.

**Table 2:** Panel of porcine oligonucleotide primers used for QPCR.

Target gene	Accession no.	Forward primer (5′–3′) Reverse primer (5′–3′)	Amplicon length (bp)
**Immune response**			
*IL6*	NM_214399.1	F: GACAAAGCCACCACCCCTAA R: CTCGTTCTGTGACTGCAGCTTATC	69
CXCL8	NM_213867.1	F: TGCACTTACTCTTGCCAGAACTG R: CAAACTGGCTGTTGCCTTCTT	82
*IL10*	NM_214041.1	F: GCCTTCGGCCCAGTGAA R: AGAGACCCGGTCAGCAACAA	71
*IL17A*	NM_001005729.1	F: CCCTGTCACTGCTGCTTCTG R: TCATGATTCCCGCCTTCAC	57
*IFNG*	NM_213948.1	F: TCTAACCTAAGAAAGCGGAAGAGAA R: TTGCAGGCAGGATGACAATTA	81
*TNF*	NM_214022.1	F: TGGCCCCTTGAGCATCA R: CGGGCTTATCTGAGGTTTGAGA	68
*TGFB1*	NM_214015.1	F: AGGGCTACCATGCCAATTTCT R: CGGGTTGTGCTGGTTGTACA	101
**Tight junctions and mucins**			
*MUC1*	XM_001926883.1	F: ACACCCATGGGCGCTATGT R: GCCTGCAGAAACCTGCTCAT	68
*MUC2*	AK231524	F: CAACGGCCTCTCCTTCTCTGT R: GCCACACTGGCCCTTTGT	70
*CLND3*	NM_001160075.1	F: GAGGGCCTGTGGATGAACTG R: GAGTCGTACACTTTGCACTGCAT	65
**Nutrient transporters**			
*FABP2*	NM_001031780.1	F:CAGCCTCGCAGACGGAACTGAA R:GTGTTCTGGGCTGTGCTCCAAGA	102
*SLC2A1 (GLUT1)*	XM_003482115.1	F:TGCTCATCAACCGCAATGA R:GTTCCGCGCAGCTTCTTC	72
*SLC2A2 (GLUT2)*	AF054835.1	F:CCAGGCCCCATCCCCTGGTT R:GCGGGTCCAGTTGCTGAATGC	96
*SLC2A5 (GLUT5)*	EU012359	F:CCCAGGAGCCGGTCAAG R:TCAGCGTCGCCAAAGCA	60
*SLC5A10 (SGLT1)*	NM_001164021.1	F: GGCTGGACGAAGTATGGTGT R: ACAACCACCCAAATCAGAGC	153
*SLC15A1(PEPT1)*	NM_214347.1	F:GGATAGCCTGTACCCCAAGCT R:CATCCTCCACGTGCTTCTTGA	73
*SLC1A4*	XM_003125088	F:ACCCTCGCCGACTTTTAGTCT R:GCCTGTGCCGAGAAGTAATCC	
**Reference genes**			
*ACTB*	XM_001927228.1	F:GGACATCGGATACCCAAGGA R:AAGTTGGAAGGCCGGTTAATTT	71
*B2M*	NM_213978.1	F:CGGAAAGCCAAATTACCTGAAC R:TCTCCCCGTTTTTCAGCAAAT	83
*GAPDH*	AF017079.1	F: CAGCAATGCCTCCTGTACCA R: ACGATGCCGAAGTTGTCATG	72
*PPIA*	NM_214353.1	F: CGGGTCCTGGCATCTTGT R: TGGCAGTGCAAATGAAAAACT	75

### Microbiological analyses

#### Microbial DNA extraction

Microbial genomic DNA was extracted using QIAamp PowerFecal Pro DNA Kit (Qiagen, West Sussex, UK) according to the manufacturer’s instructions. The DNA quantity and quality were evaluated using a Nanodrop ND-1000 Spectrophotometer (Thermo Scientific, Wilmington, DE).

#### Illumina sequencing

Extraction of the bacterial DNA from the caecal digesta samples and high-throughput sequencing of the V3–V4 hypervariable region of the bacterial 16S rRNA gene was performed on an Illumina MiSeq platform according to their standard protocols (Eurofins Genomics, Ebersberg, Germany). Briefly, the V3–V4 region was PCR-amplified using universal primers containing adapter overhang nucleotide sequences for forward and reverse index primers. Amplicons were purified using AMPure XP beads (Beckman Coulter, Indianapolis, IN) and set up for the index PCR with Nextera XT index primers (Illumina, San Diego, CA). The indexed samples were purified using AMPure XP beads, quantified using a fragment analyser (Agilent, Santa Clara, CA), and equal quantities from each sample were pooled. The resulting pooled library was quantified using the Bioanalyzer 7500 DNA kit (Agilent, Santa Clara, CA) and sequenced using the v3 chemistry (2 × 300 bp paired-end reads).

#### Bioinformatic

The bioinformatic analysis of the resulting sequences was performed by Eurofins Genomics (Ebersberg, Germany) using the open-source software package (version 1.9.1) Quantitative Insights into Microbial Ecology (QIIME)^19^. All raw reads passing the standard Illumina chastity filter were demultiplexed according to their index sequences (read quality score > 30). The primer sequences were clipped from the starts of the raw forward and reverse reads. If primer sequences were not perfectly matched, read pairs were removed to retain only high-quality reads. Paired-end reads were then merged if possible, to obtain a single, longer read that covers the full target region using the software FLASH 2.2.00^20^. Pairs were merged with a minimum overlap size of 10 bp to reduce false-positive merges. The forward read was only retained for the subsequent analysis steps when merging was not possible. Merged reads were quality filtered according to the expected length and known length variations of the V3–V4 region (ca. 445 bp). The ends of retained forward reads were clipped to a total read length of 285 bp to remove low quality bases. Merged and retained reads containing ambiguous bases were discarded. The filtered reads (merged and quality clipped retained forward reads) were used for the microbiome profiling. Chimeric reads were identified and removed based on the de-novo algorithm of UCHIME^21^ as implemented in the VSEARCH package^22^. The remaining set of high-quality reads were processed using minimum entropy decomposition (MED) to partition reads to operational taxonomic units (OTU)^23,24^. DC-MEGABLAST alignments of cluster representative sequences to the NCBI nucleotide sequence database were performed for taxonomic assignment (from phylum to species) of each OTU. A sequence identity of 70% across at least 80% of the representative sequence was the minimal requirement for considering reference sequences. Abundances of bacterial taxonomic units were normalized using lineage-specific copy numbers of the relevant marker genes to improve estimates^25^.

The normalized OTU table combined with the phenotype metadata and phylogenetic tree comprised the data matrix. This matrix was then input into the phyloseq package within R (http://www.r-project.org; version 3.5.0). The dynamics of richness and diversity in the microbiota were computed with the observed, Chao1, ACE, Shannon, Simpson, InvSimpson and Fisher indices. The Simpson and Shannon indices of diversity account for both richness and evenness parameters. The beta diversity measurements are a measure of separation of the phylogenetic structure of the OTU in one sample compared with all other samples. This was estimated by normalising the data so taxonomic feature counts were comparable across samples. Several distance metrics were considered, to calculate the distance matrix of the different multidimensional reduction methods. These included weighted/unweighted UniFrac distance and non-phylogenetic distance metrics (i.e., Bray–Curtis, Jensen–Shannon divergence and Euclidian) using phyloseq in R^26,27^. Differential abundance testing was performed on tables extracted from the phyloseq object at phylum, family, and genus level. The data was analysed using the PROC Glimmix procedure within Statistical Analysis Software (SAS) 9.4 (SAS Institute, Cary, NC, USA). The model assessed the effect of ‘treatment’, with the piglet (sow pen) as the experimental unit. 10 sows and 10 piglets per treatment group were used for the statistical analysis of the relative bacterial abundances. Results are presented using Benjamini–Hochberg (BH) adjusted P-values.

### Feed analysis

The feed samples were milled through a 1 mm screen (Christy and Norris Hammer Mill, Chelmsford, England) and retained for chemical analysis. The gross energy (GE) content was determined using an adiabatic bomb calorimeter (Parr Instruments, Moline, IL USA) as previously described^28^. The dry matter (DM) content of the feed was determined after drying overnight at 104 °C. Feed samples were analysed for crude ash (AOAC method 942.05^29^) and nitrogen (N × 6.25; AOAC method 990.03^29^). All samples were measured in duplicate.

### Statistical analysis

The data on performance, intestinal morphology and gene expression were initially checked for normality using the univariate procedure of Statistical Analysis Software^30^. The data on performance, intestinal morphology, and gene expression were analysed using the GLM procedure within SAS. The model assessed the effect of treatment, with the sow as the experimental unit. For piglet diarrhoea score, the data was analysed by repeated-measures analysis using the PROC MIXED procedure of SAS. All data presented in the tables are expressed as least-square means with their standard errors. The probability level that denoted significance was P < 0.05. Data are presented as least-square means and standard error of the mean.

### Ethics statement

All experimental procedures described in the present experiment were approved under the University College Dublin Animal Research Ethics Committee, Ireland (AREC-17-38-Sweeney). The study was conducted in accordance with Irish legislation (SI no. 543/2012) and the EU directive 2010/63/EU for animal experimentation and in compliance with the ARRIVE guidelines. The authors confirm that the ethical policies of the journal, as noted on the journal’s author guidelines page, have been adhered to and the appropriate ethical review committee approval has been received. The authors confirm that they have followed the EU standards for the protection of animals used for scientific purposes and the ARRIVE guidelines.

## Results

### Gestation length and suckling piglet growth performance

The effect of maternal supplementation on gestation length and piglet body weight and ADG is presented in Table 3. There was no effect of maternal dietary supplementation on gestation length (115.2 vs 115.4 days, P = 0.724) and piglet body weight at birth (1.23 vs 1.15 kg, P = 0.052) and weaning (6.46 vs 6.58, P = 0.721), and ADG (0.19 vs 0.19, P = 0.536) from birth to weaning (P > 0.05).

**Table 3:** The effect of maternal dietary treatment on gestation length and piglet performance at birth and weaning (least square means with their standard errors).

	Control*	Supplemented*	SEM	P-values
Number of sows	10	10		
Gestation length	115.2	115.4	0.543	0.724
Birth weight (kg)	1.23	1.15	0.054	0.052
Weaning weight (kg)	6.46	6.58	0.231	0.721
Average daily gain (kg)	0.19	0.19	0.012	0.536

Supplemented = Casein hydrolysate + yeast β-glucan, and control = basal lactation diet.

### The effects of maternal dietary supplementation on the sow faecal and piglet caecal and colonic microbiota

#### Bacterial richness and diversity

**Figure 1:**
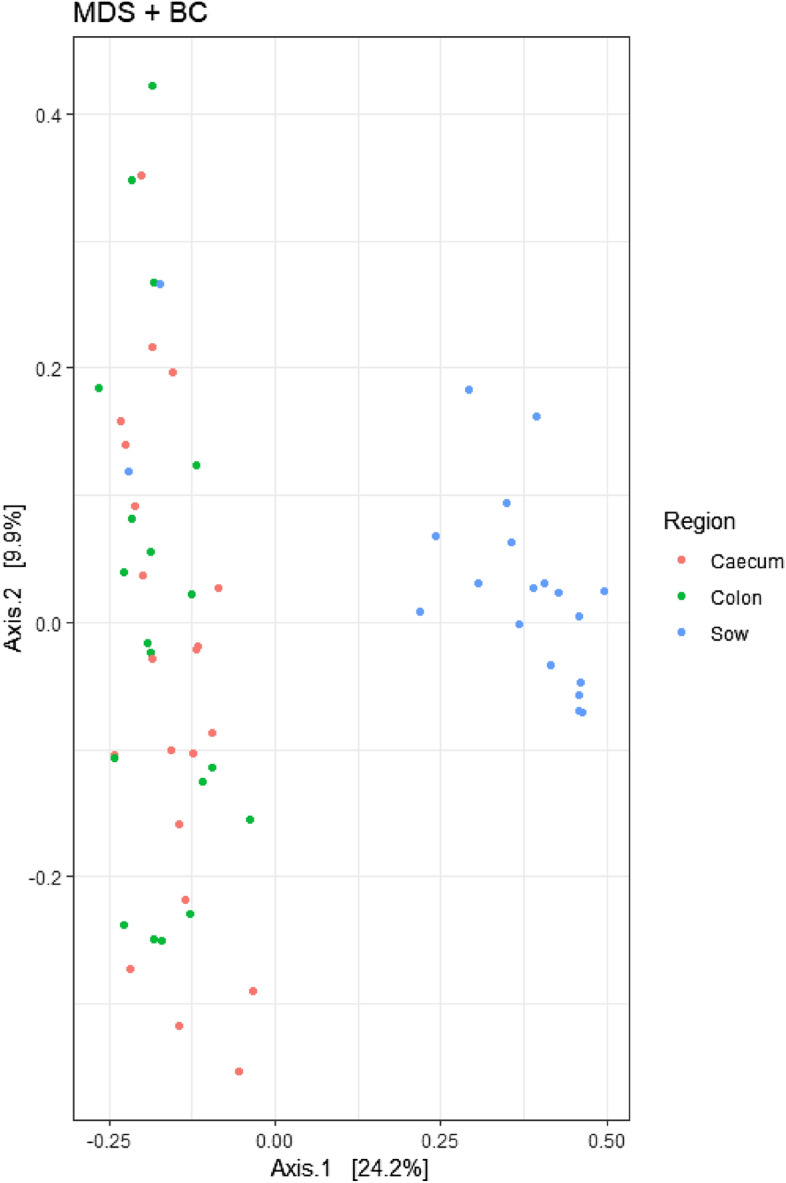
Bacterial beta diversity in piglet colon and caecum and faeces based on Permanova analysis and through visualisation using the Bray Curtis distance matrix and multi-dimensional scaling.

The effects of maternal supplementation on the measures of beta diversity and alpha diversity are presented in Fig. 1 and Table 4, respectively. Beta diversity in the piglets caecal and colonic microbiome differed to that of the sow (P < 0.05) based on Permanova analysis and through visualisation using the Bray Curtis distance matrix and multi-dimensional scaling. Maternal dietary supplementation had no effect on the Observed, Chao1, ACE, Shannon, Simpson, InvSimpson and Fisher index measures of alpha diversity (P > 0.05) in either the sows faeces or the piglets caecal and colonic digesta.

**Table 4:** The effect of dietary treatment on measures of alpha diversity (least-square means with their standard errors).

	Control	Supplemented	SEM	P value
**Sow faeces**^**a**^				
Observed	151.6	150.0	27.70	> 0.05
Chao1	152.4	150.6	28.34	> 0.05
Fisher	29.8	29.4	6.71	> 0.05
Shannon	4.0	4.0	0.36	> 0.05
Simpson	1.0	1.0	0.02	> 0.05
**Piglet caecum**^**b**^				
Observed	126.8	139.1	25.33	> 0.05
Chao1	127.7	140.1	25.89	> 0.05
Fisher	23.8	26.9	5.97	> 0.05
Shannon	3.6	3.6	0.30	> 0.05
Simpson	0.9	0.9	0.02	> 0.05
**Piglet colon**^**b**^				
Observed	129.6	132.3	29.69	> 0.05
Chao1	131.5	134.1	31.05	> 0.05
Fisher	24.7	25.2	6.87	> 0.05
Shannon	3.6	3.7	0.24	> 0.05
Simpson	0.9	0.9	0.02	> 0.05

Supplemented = casein hydrolysate + yeast β-glucan supplementation, and control = basal lactation diet.

^a^Ten sows per treatment group.

^b^10 piglets per treatment group.

#### Differential bacterial abundance analysis

All data on bacterial abundances at phylum, family and genus level are provided in Tables 5 and 6.

**Table 5:** The effect of maternal dietary treatment on the % bacterial abundance at phylum and family level (Least-square means with their standard errors).

	Control	Supplemented	SEM	P-values
**Phylum**				
Sow faeces*				
Firmicutes	82.27	93.11	2.982	0.019
Proteobacteria	11.34	1.69	0.724	< 0.001
Piglet caecum^¥^				
Actinobacteria	10.1	5.67	0.86	0.002
Verrucomicrobia	1.01	0.08	0.203	0.031
Piglet colon^¥^				
Actinobacteria	8.25	5.06	0.831	0.024
**Family**				
Sow faeces*				
Lactobacillaceae	5.04	21.34	1.108	< 0.001
Lachnospiraceae	14.06	18.73	1.286	0.019
Christensenellaceae	12.31	8.93	1.027	0.035
Moraxellaceae	9.07	0.55	0.578	< 0.001
Planococcaceae	1.51	0.35	0.283	0.028
Piglet caecum^¥^				
Lactobacillaceae	18.09	23.48	1.403	0.015
Ruminococcaceae	14.66	10.27	1.088	0.01
Coriobacteriaceae	7.54	4.97	0.77	0.03
Erysipelotrichaceae	3.94	6.62	0.702	0.017
Atopobiaceae	5.49	0.9	0.514	< 0.001
Christensenellaceae	1.45	3.8	0.484	0.005
Oscillospiraceae	0.82	2.32	0.373	0.018
Akkermansiaceae	1.59	0.13	0.254	0.009
Piglet colon^¥^				
Erysipelotrichaceae	3.78	6.06	0.718	0.039
Atopobiaceae	1.06	4.53	0.518	< 0.001
Christensenellaceae	4.24	0.7	0.464	< 0.001

* Ten sows per treatment group. ¥ 10 piglets per treatment group.

**Table 6:** The effect of maternal dietary treatment on the % bacterial abundance at genus level (Least-square means with their standard errors).

Genus	Control	Supplemented	SEM	P-values
**Sow faeces**^**a**^				
Lactobacillus	4.97	21.08	1.102	< 0.001
Christensenella	11.13	7.50	0.959	0.018
Acinetobacter	9.43	0.55	0.586	< 0.001
Metabacterium	0.30	4.20	0.424	< 0.001
Kineothrix	3.53	1.42	0.482	0.012
Niameybacter	0.11	1.77	0.272	0.009
Gemmiger	0.06	1.69	0.252	0.017
**Piglet caecum**^**b**^				
Lactobacillus	17.80	23.61	1.400	0.009
Collinsella	7.55	5.20	0.778	0.046
Gemmiger	9.35	4.18	0.792	< 0.001
Holdemania	0.96	5.23	0.500	< 0.001
Oscillibacter	0.83	2.48	0.382	0.013
Christensenella	0.75	2.46	0.373	0.009
Flavonifractor	0.30	2.36	0.318	0.003
Intestinimonas	2.30	0.95	0.387	0.029
Olsenella	5.69	0.91	0.521	< 0.001
Ruminococcus	1.68	0.63	0.324	0.043
**Piglet colon**^**b**^				
Gemmiger	7.03	1.95	0.652	< 0.001
Holdemania	0.78	4.48	0.493	< 0.001
Ruminococcus	2.51	4.46	0.602	0.038
Olsenella	4.18	0.69	0.462	< 0.001
Flavonifractor	0.35	3.34	0.398	< 0.001
Christensenella	0.52	2.90	0.398	0.002

Supplemented = Casein hydrolysate + yeast β-glucan supplementation, and control = basal lactation diet.

^a^Ten sows per treatment group.

^b^10 piglets per treatment group.

#### Sow faeces at farrowing

##### Phylum

There were six bacterial phyla identified with Firmicutes being the dominant phyla (~ 87.69%) followed by Proteobacteria (~ 6.52%), Spirochaetes (~ 3.50%), Actinobacteria (~ 1.38%), Tenericutes (~ 0.47%), and Synergistetes (~ 0.10%). Firmicutes were increased (P < 0.05) and Proteobacteria were decreased (P < 0.05) in the supplemented sows compared to the control sows.

##### Family

At family level, dietary supplementation increased the relative abundance of *Lactobacillaceae* (P < 0.0001) and *Lachnospiraceae* (P < 0.05) compared with the control group. Dietary supplementation decreased the relative abundance of *Christensenellaceae* (P < 0.05), *Planococcaceae* (P < 0.05) and *Moraxellaceae* (P < 0.0001) compared to the control.

##### Genus

At genus level, dietary supplementation increased the relative abundance of *Lactobacillus* (P < 0.001), *Metabacterium* (P < 0.01), *Niameybacter* (P < 0.01) and *Gemmiger* (P < 0.05) compared to the control. Dietary supplementation decreased the relative abundance of *Christensenella* (P < 0.05), *Kineothrix* (P < 0.05) and *Acinetobacter* (P < 0.0001) compared to the control.

#### Piglets caecal and colonic microbiota

##### Phylum

In the caecum and colon, there were six bacterial phyla identified with Firmicutes being the dominant phyla followed by Actinobacteria, Proteobacteria, Verrucomicrobia, Fusobacteria, and Bacteroidetes. The relative abundance of Actinobacteria was decreased (P < 0.05) in the caecum and colon of piglets from supplemented sows compared to piglets from control sows.

##### Family

In the caecum, the relative abundance of *Lactobacillacea* and *Oscillospiraceae* were increased (P < 0.05), while the relative abundance of *Coriobacteriaceae*, *Ruminococcaceae* and *Akkermansiaceae* decreased (P < 0.05) in piglets from supplemented sows compared to piglets from control sows. In the caecum and colon, the relative abundance of *Erysipelotrichaceae* and *Christensenellaceae* were increased (P < 0.05), while the relative abundance of *Atopobiaceae* was decreased (P < 0.05) in piglets from supplemented sows compared to piglets from control sows.

### Gene expression in the small intestine

The intestinal expression of genes related to nutrient digestion and absorption, mucosal barrier function, and immunity in the duodenum, jejunum and ileum of piglets are presented in Table 7.

**Table 7:** The effects of maternal dietary supplementation on the expression of nutrient transporter, immune marker and tight junction genes in piglet’s duodenum, jejunum and ileum (least square means with their standard errors).

	Gene	Control	Supplemented	SEM	P-value
Duodenum^*^					
Nutrient transporters	SLC15A1	0.88	1.66	0.175	0.005
	FABP2	0.89	1.40	0.128	0.010
	SLC2A1	1.04	1.78	0.253	0.052
	SLC2A5	0.94	2.66	0.522	0.032
	SLC1A4	2.62	0.75	0.501	0.017
Tight junctions and immune markers	CLDN3	0.82	1.81	0.211	0.004
	MUC2	0.84	1.55	0.166	0.007
	IL6	4.34	0.57	1.017	0.017
	TNF	1.83	0.86	0.327	0.051
	TGFB	1.53	0.83	0.207	0.026
Jejunum^*^					
Nutrient transporters	FABP2	1.07	2.05	0.245	0.011
	SLC15A1	0.85	2.80	0.482	0.010
	SGLT1	0.76	2.00	0.295	0.008
	SLC16A	0.82	1.50	0.249	0.072
	SLC1A4	2.58	0.69	0.358	0.002
	SLC5A8	1.18	2.28	0.378	0.055
	SLC2A1	0.66	1.17	0.133	0.015
	SLC2A2	1.00	2.80	0.453	0.012
	SLC2A5	0.67	1.99	0.257	0.002
Tight junctions and immune markers	Claudin 3	0.53	2.37	0.204	< 0.0001
	MUC2	0.67	2.69	0.347	0.001
	IL10	1.73	0.81	0.215	0.007
	IL6	2.63	0.64	0.294	0.000
	TGFB	1.46	0.82	0.176	0.019
	TNF	1.72	0.78	0.184	0.002
	CXCL8	0.58	2.03	0.249	0.001
Ileum^*^					
Tight junctions and immune markers	IFNG	1.58	0.88	0.224	0.043

SLC15A1/PEPT1, peptide transporter 1; FABP2, fatty acid binding protein 2; SLC2A1/GLUT1, glucose transporter 1; SLC2A2/GLUT2, glucose transporter 2; SLC2A5/GLUT5, glucose transporter 5; SLC5A1/SGLT1, sodium glucose linked transporter 1; SLC5A8, solute carrier family 5 member 8; SLC16A, solute carrier family 16 member 1; SLC1A4, solute carrier family 1 member 4; TNF, tumor necrosis factor alpha; CXCL8, interleukin 8; IL6, interleukin 6; IL10, interleukin 10; IFNG, interferon gamma; MUC2, mucin-2; TGF-β, transforming growth factor beta; CLDN3, claudin 3;

*A total of 10 replicates were used per treatment.

#### Nutrient transporter gene expression

The gene expression of *FABP2* (fatty acid binding protein 2, P < 0.01), *SLC15A1* (peptide transporter 1, P < 0.01), *SLC2A2* (glucose transporter 2, P < 0.05), *SLC2A5* (glucose transporter 5, P < 0.05) and *SLC1A4* (solute carrier family 1 member 4, P < 0.05) was upregulated in the duodenum and jejunum of piglets suckling the supplemented sows compared with those suckling the control sows. In the jejunum, the gene expression of *SLC5A1* (sodium transporter 1, P < 0.01) was upregulated and there was a numerical tendency for *SLC16A* (solute carrier family 16 member 1, P < 0.07) and *SLC5A8* (solute carrier family 5 member 8, P < 0.07) to be upregulated in piglets suckling the supplemented sows compared with those suckling the control sows.

#### Expression of genes involved in inflammation and the epithelial barrier

The expression of *IL6* (interleukin-6, P < 0.05), *TNF* (tumor necrosis factor, P < 0.05) and *TGFB* (transforming growth factor beta-1, P < 0.05) were down-regulated in the duodenum and jejunum of piglets suckling the supplemented sows compared with those suckling the control sows. The expression of *CLDN3* (claudin-3, P < 0.01) was up-regulated in the duodenum and jejunum of piglets suckling the supplemented sows compared with those suckling the control sows. The expression of *IL10* (interleukin-10, P < 0.01) was down-regulated in the jejunum of piglets suckling the supplemented sows compared with those suckling the control sows. The expression of *CXCL8* (interleukin 8, P < 0.01) and *CLDN3* (P < 0.0001) were upregulated in the jejunum of piglets suckling the supplemented sows compared with those suckling the control sows.

#### Ileum

The expression of *IFN* (interferon gamma, P < 0.05) was downregulated in the ileum of piglets suckling the supplemented sows compared with those suckling the control sows.

### Piglet small-intestinal morphology

The effect of maternal supplementation on piglet small intestinal morphology is presented in Table 8.

**Table 8:** Effect of maternal dietary supplementation on villus height and crypt depth in the small intestine (least square means with their standard errors).

	Control*	Supplemented*	SEM	P -values
**Duodenum**				
VH µm	318.2	402.9	28.07	0.046
CD µm	97.3	117.7	5.13	0.012
VH:CD	3.3	3.4	0.27	0.754
**Jejunum**				
VH µm	269.4	281.2	12.21	0.506
CD µm	83.3	89.8	3.29	0.182
VH:CD	3.4	3.2	0.32	0.674
**Ileum**				
VH µm	232.5	244.5	8.87	0.351
CD µm	91.3	79.4	4.18	0.068
VH:CD	2.7	3.1	0.12	0.022

*VH* villus height, *CD* crypt depth, *VH:CD* villus height to crypt depth ratio.

Supplemented = casein hydrolysate + yeast β-glucan supplementation, and control = basal lactation diet.

*A total of 10 replicates were used per treatment group.

In the duodenum, piglets suckling the supplemented sows had larger VH (P < 0.05) compared with those suckling the control sows. In the ileum, piglets suckling supplemented sows had a larger VH:CD ratio (P < 0.05) compared with those suckling the control sows.

## Discussion

In the present study, it was hypothesised that maternal supplementation with a casein hydrolysate and yeast β-glucan (CH-YBG) from day 83 of gestation would alter the GIT microbiota of the sow and modulate selected intestinal microbial populations, the inflammatory response, and aspects of intestinal health of her progeny at weaning. Maternal supplementation with CH-YBG altered the sow’s faecal microbiota by increasing the relative abundance of bacterial members of the Firmicutes phylum, including *Lactobacillus.* The positive response observed in piglets suckling the CH-YBG supplemented sows, such as increased abundance of numerous Firmicutes, including *Lactobacillus* and *Christensenella* in the caecal microbiota, improved intestinal morphology, decreased expression of proinflammatory cytokine genes, and increased expression of nutrient transporter, tight junction, and mucin genes at the time of weaning supports this hypothesis.

The early colonization of the piglets intestinal microbiota plays an essential role in the development of the piglets intestinal immune system, barrier function and nutrient absorption^31,32^. The major microbial changes in this experiment were observed within the Firmicutes, with supplemented sows having a higher abundance of beneficial bacteria such as *Lactobacillus* compared to the control sows. The abundance of *Lactobacillus, Christensenella* and *Erysipelotrichaceae* was also increased in the progeny of supplemented sows at 28 days of age. These genera are positively correlated with a healthy gut, with low numbers associated with inflammatory disorders such as inflammatory bowel disease^33,34^ and Crohns disease^35,36^. The increase in *Lactobacillus* in the sow is likely to be attributable to the casein hydrolysate’s role as a substrate for the growth of Lactobacilli, as casein hydrolysate supplementation increased the growth of Lactobacillus in rats and pigs^37,38^. Both *Lactobacillus* and *Christensenella* species have strong anti-inflammatory properties and play an important role in maintaining microbial symbiosis, thereby contributing to the establishment of an “optimal” microbiota in piglets at weaning. These results indicate that maternal supplementation positively influenced the microbial composition in the faeces of the sow and in the large intestine of her progeny and thus, may play a role in improving the health of piglets at weaning.

The small intestine plays an essential role in the digestion and absorption of nutrients and serves as a key regulator of growth. Under conventional practice, abrupt weaning is often associated with undesirable morphological changes, such as villous atrophy and crypt hyperplasia, leading to functional changes such as a decrease in nutrient digestion and absorption^2,39^. Thus, supplements that positively influence intestinal health should aid in the amelioration of the intestinal dysfunction associated with weaning stress. Supplementation with bacterial members of the genus *Lactobacillus* has been shown to improve intestinal health in broilers through the improvement of small intestinal villous height and crypt depth^40,41^. In the present study, pigs weaned from supplemented sows had improved VH in the duodenum and VH to CD ratio in the ileum.

The improvement in intestinal health in response to maternal supplementation, consequently enhanced the digestive and absorptive capacity of the intestinal epithelium resulting in an upregulation of nutrient transporters in the small intestine. Maternal dietary supplementation with CH-YBG increased the expression of fatty acid transporter *FABP2*, peptide transporter *SLC15A1*, glucose transporters *SLC2A1*, *SLC2A2*, *SLC2A5*, and *SGLT1*, amino acid transporter *SLC1A4*, and sodium transporter *SLC5A8* and had a tendency to increase solute carrier *SLC16A*. This suggests there is increased availability and transport of fatty acids, sugars, sodium, peptides and amino acids in the duodenum and jejunum of pigs suckling supplemented sows. The upregulation of these nutrient transporters indicates a positive functional adaptation in the pigs, and potentially improving their ability to digest a diet primarily comprised of carbohydrates and protein post-weaning.

In the present study, maternal supplementation increased the expression of the tight junction gene, *CLDN3* and the mucin gene *MUC2.* Claudin 3 is a tight junction integral protein that decreases paracellular permeability, thus preventing the translocation of luminal antigens^42^. Mucins provide a barrier against potential pathogens while also modulating the expression of inflammatory cytokines^43^. It is possible that the increased expression of *CLDN3* reduced the translocation of bacteria across the intestinal barrier, while the increased expression of *MUC2* reduced the adherence of bacteria to the intestinal cells, thereby reducing the risk of pigs to infection and diarrhoea. The increase in genes associated with intestinal barrier function was concomitant with a decrease in the expression of pro-inflammatory cytokine genes. Overall, these effects indicate a better host immune response with regard to potential environmental or pathogenic challenge at weaning time. Maternal supplementation from day 83 of gestation and throughout the lactation period decreased expression of the pro-inflammatory cytokine genes *IL6*, *TGFB*, *TNF* and *IFNG*. IL-6 and TNF are potent mediators of inflammation and play an important role in the pathogenesis of E. coli caused diarrhoea^44,45^. It is likely that the increase in the expression of the tight junction genes strengthened the intestinal barrier, thus limiting the ability of antigens to enter the body and prevent adverse immune responses. When the immune system is activated, and inflammation occurs, maintenance energy and amino acid requirements are increased, thereby reducing the proportion of daily nutrient intake directed towards growth^46^. Thus, the potential decrease in immune system activity in maternally supplemented pigs may result in an improvement in growth performance during the post-weaning period.

While it was not within the scope of this study to identify what effects each bioactive had on the gastrointestinal health parameters measured, it is worthy to note that the 5 kDa retentate (5kDaR) of a CH has established microbial activity and anti-inflammatory effects in vitro^9,47,48^. Furthermore, in a previous study the combination of the 5kDaR + YBG positively enhanced gastrointestinal parameters in pigs post-weaning, whereas the individual inclusion of either 5kDaR or YBG were not effective^10^. It is evident in the present study that maternal supplementation with a CH-YBG improved several gastrointestinal health parameters that influence performance. Thus, it may be anticipated that this would translate into an improvement in growth performance at weaning, however piglet weights at weaning were not influenced by sow diet. A similar effect was observed following maternal supplementation with seaweed-derived polysaccharides, containing laminarin and fucoidan^49^. Furthermore, maternal supplementation with *Bacillus altitudinis*^50^ did not influence the growth performance of piglets at weaning, however maternal supplementation had an impact on the progeny’s performance post-weaning. A further study to explore the effects of maternal supplementation with a CH-YBG on piglet growth performance post-weaning is warranted.

## Conclusion

Maternal dietary supplementation with a combination of a casein hydrolysate + yeast β-glucan from day 83 of gestation to weaning had a positive effect on the microbial population of sows and their piglets, represented by a higher abundance of several bacterial members of the phylum Firmicutes, including *Lactobacillus* and *Christensenella*. Maternal supplementation enhanced piglet gastrointestinal health by improving intestinal morphology, reducing the expression of proinflammatory cytokine genes, and increasing the expression of nutrient transporter, tight junction, and mucin genes at weaning. These findings indicate that maternal casein hydrolysate + yeast β-glucan supplementation may be a promising strategy for improving the gastrointestinal health and function of piglets at weaning, making piglets more resilient to post-weaning challenges.

## Data Availability

The datasets used and/or analysed during the current study are available from the corresponding author on reasonable request.
